# Clinical outcomes of patients with gastrointestinal stromal tumor in phase I clinical trials

**DOI:** 10.1186/s12885-016-2939-0

**Published:** 2016-11-14

**Authors:** Yoshiaki Nagatani, Kohei Shitara, Hideaki Bando, Yasutoshi Kuboki, Wataru Okamoto, Takashi Kojima, Takayuki Yoshino, Toshirou Nishida, Atushi Ohtsu, Toshihiko Doi

**Affiliations:** 1 Department of Gastroenterology and Gastrointestinal Oncology, National Cancer Center Hospital East (NCCHE), 6-5-1 Kashiwanoha, Kashiwa, Chiba 277-8577 Japan; 2 Department of Surgery, National Cancer Center Hospital East (NCCHE), 6-5-1 Kashiwanoha, Kashiwa, Chiba 277-8577 Japan

**Keywords:** Gastrointestinal stromal tumor, Phase I clinical trial, Systemic chemotherapy, Investigational agent

## Abstract

**Background:**

The prognosis of patients with gastrointestinal stromal tumor (GIST) after the failure of standard therapies is poor with supportive care alone. Guidelines recommend clinical trials, and patients with good performance status following standard therapies are often eligible for phase I clinical trials of investigational agents; however, there are no detailed reports on the clinical outcomes of GIST patients enrolled in these trials.

**Methods:**

We retrospectively reviewed the clinical outcomes of 21 consecutive GIST patients who were enrolled in one or more phase I clinical trials at a single center between March 2009 and November 2014.

**Results:**

The median age was 57 years, and the median number of previous lines of standard chemotherapy was three. Chemotherapy before enrollment in a phase I clinical trial included imatinib, sunitinib, and regorafenib in 100, 95, and 43 % of patients, respectively. None of the patients achieved objective response. Ten patients (47.6 %) were determined to be stable according to the Response Evaluation Criteria in Solid Tumors; four of them (19.0 %) maintained their status for more than 24 weeks. Four patients achieved partial response according to the Choi criteria. No dose-limiting toxicity was observed; however, severe adverse events and grade 3 or higher toxicities were reported in one (4.8 %) and two patients (9.5 %), respectively. Although no treatment-related deaths occurred, one patient (4.8 %) died within 30 days after the last drug administration because of disease progression. The median progression-free survival was 1.9 months, and the median overall survival time has not been reached.

**Conclusions:**

Data suggested that phase I clinical trials were feasible and may provide prognostic benefits to GIST patients after standard therapies, indicating that enrollment in these studies may provide a treatment option for these patients.

## Background

Gastrointestinal stromal tumors (GISTs) are rare tumors of the gastrointestinal tract, with an estimated number of new cases per year being 4000–6000 in the United States [1]; such tumors are predicted to affect approximately two cases per 100,000 individuals per year in Japan [2]. Approximately 90 % of GISTs have a mutation in *c-kit* or platelet-derived growth factor receptor alpha (*PDGFRA*), which leads to constitutive activation of the tyrosine kinase activity of KIT or PDGFRA as the main carcinogenic mechanism of GIST, respectively [3, 4]. The remaining 10 % of GISTs are not associated with these genomic alterations and are considered as the wild-type GISTs [5, 6].

Imatinib, an oral receptor tyrosine kinase inhibitor (TKI), is the first-line systemic chemotherapeutic agent for metastatic or unresectable GIST [7–9]. Sunitinib is an oral multi-target receptor TKI that mainly binds to vascular endothelial growth factor receptors (VEGFRs) 1, 2, and 3; its use improves the time to progression (TTP) compared with the use of placebo after treatment failure with imatinib (median TTP: 6.4 weeks with placebo vs. 27.3 weeks with sunitinib) [10–13]. Recently, the use of regorafenib, which targets kinases involved in angiogenesis, oncogenesis (i.e., KIT, Ret, and BRAF), and tumor microenvironment [i.e., PDGFR and fibroblast growth factor receptor (FGFR)], showed a statistically significant improvement in progression-free survival (PFS) compared with the use of placebo (median PFS, 4.8 and 0.9 months, respectively) [14]. Despite these TKIs, the prognosis of GIST patients after the failure of standard chemotherapy is still poor because reported median PFS is less than 1 month with supportive care (BSC) alone [14, 15]. Treatment options for these patients with a good performance status (PS) may include clinical trials of investigational agents, TKI rechallenge, and TKI continuation beyond progression according to the guidelines of the National Comprehensive Cancer Network (NCCN), the European Society for Medical Oncology (ESMO) group [16, 17]. Similar recommendation was also reported in the Japanese guideline [18]. Disease-specific phase II or III trials may be preferred options than all-comer phase I trials, if available, because the primary objectives of phase I clinical trials are to evaluate the safety and determine the maximum-tolerated dose (MTD), pharmacokinetics (PK), and pharmacodynamics (PD) [19]. However, disease-specific phase II or III trials are not always available for rare cancers such as GIST. The safety and exploratory efficacy analyses of phase I clinical trials have been reported in some malignancies [20, 21]; however, the clinical outcome of GIST patients in the all-comer phase I clinical trials has not yet been reported. Here we retrospectively evaluated the safety as well as exploratory efficacy of phase I clinical trials in GIST patients at our institution to rationalize the enrollment of GIST patients in phase I clinical trials which was one of treatment options after failure of the standard therapy.

## Methods

### Patients

We reviewed the electronic medical records of 21 consecutive GIST patients who were enrolled in phase I clinical trials after failure of systemic chemotherapy at our institution between March 2009 and November 2014. All patients having histologically-proven metastatic/recurrent GIST diagnosed by a pathologist at our institution had received standard chemotherapy at that time and had experienced refractory diseases. Although the phase I clinical trials open for GIST patients have changed over time according to the trial availability at our institution and the eligibility criteria, we proposed the phase I clinical trials as one of therapeutic options for the patients who finished standard chemotherapy and could not be enrolled in phase II or III trials for GIST because of availability. We proposed phase I clinical trials among the trials being conducted with respect to KIT/PDGFRA inhibitors, drugs inhibiting downstream kinases of KIT and PDGFRA tyrosine kinases, angiogenesis inhibitors, drugs which have some evidences in sarcoma, and others. Finally, patients were selected when they fulfilled the eligibility criteria. This retrospective study was conducted under the approval of the institutional review board according to the Japanese ethical guidelines for epidemiologic research. Written informed consent was obtained from all patients before enrollment in each phase I clinical trial.

### Methods and treatment evaluation

The following baseline characteristics were collected from each patient: age, gender, Eastern Cooperative Oncology Group (ECOG) PS, primary site, site of metastasis, number of metastases, number of previous lines of chemotherapy, previous treatment, and number and type of enrolled phase I clinical trials. Safety profiles during each phase I clinical trial, including dose-limiting toxicity (DLT), severe adverse events (SAEs) as defined in each protocol, grade 3 or higher toxicities, and death within 30 days after the last drug administration were evaluated in accordance with each protocol using the Common Terminology Criteria for Adverse Events version 3.0 or 4.0. Tumor response was assessed by each investigator based on the Response Evaluation Criteria in Solid Tumors (RECIST) version 1.0 or 1.1 in each protocol. Response according to the Choi criteria, which considers changes in both size and density of the tumor based on computed tomography (CT), [21] was also retrospectively assessed by two investigators (YN and KS). Partial response (PR) according to the Choi criteria was defined as a 10 % decrease in size or a 15 % decrease in tumor density (HU) based on enhanced CT without the appearance of new lesions or obvious progression of nonmeasurable disease. If patients underwent positron emission tomography (PET) during phase I clinical trials, response was also retrospectively evaluated according to the PET response criteria by European Organization for Research and Treatment of Cancer (EORTC) [22]. Information about the date of treatment discontinuation, disease progression, last follow-up visit, and death were also collected.

### Statistical analysis

Objective response rate (ORR) was defined as the percentage of patients who achieved complete response (CR) or PR. Disease control rate (DCR) was defined as the percentage of patients who achieved CR, PR, or stable disease (SD) more than 24 weeks. PFS was defined as the time from the start of treatment in a given phase I clinical trial to disease progression or death from any cause. Best PFS for each individual was defined as the longest PFS in patients enrolled in more than one clinical trial. OS was defined as the time from enrollment in a phase I clinical trial to death from any cause. Patients who were continuing the treatment or were lost to follow-up were censored for PFS at the last follow-up when they were known to be alive and free from disease progression. Patients who were not dead or lost to follow-up were censored for OS at the time when they were last known to be alive. The median PFS and OS were estimated by the Kaplan–Meier method. PFS of subgroups according to response according to the RECIST or Choi criteria and the number of previous lines of chemotherapy were also compared using the log-rank test. Hazard ratios (HRs) were calculated using the Cox proportional hazards model with a single covariate.

In comparison of the two sides, a *P* value of <0.05 was considered statistically significant. The decision to discontinue treatment based on protocol was made by the treating physician and based on the patient’s history, clinical presentation, and imaging studies (response assessment using the RECIST criteria). All statistical analyses were performed using IBM SPSS Statistics version 21 (IBM Corporation, Armonk, NY, USA).

## Results

### Patient characteristics

Twenty-one consecutive GIST patients participated in 16 phase I clinical trials between March 2009 and November 2014 at our institution and were included in this analysis. The median length of follow-up was 0.6 years (range 0.1–5.1) for all patients. A summary of baseline patient characteristics is displayed in Table 1. The median age was 57 years (range, 29–77), and 14 patients (66.7 %) were males. Nineteen patients (90.5 %) were PS 0 and two (9.5 %) were PS 1 according to the ECOG criteria. The small intestine (47.6 %) was the most common primary site, followed by the stomach (33.3 %). The median number of previous lines of approved standard chemotherapy was three (range, 2–5). Imatinib, sunitinib, and regorafenib were administrated in 100, 95.2, and 42.9 % of patients, respectively. Genotype information was available only for four patients, two had *KIT* exon 11 mutations and the other two were wild-type GIST; genotypes of the remaining 17 patients were unavailable. The median interval from initiation of the first-line chemotherapy to the enrollment for phase I clinical trial was 4.5 years (range, 1.4–10.1). Nine patients (42.9 %) were enrolled in two or more phase I clinical trials. In the phase I clinical trials, 19 patients (90.5 %) were treated with an investigational molecular targeted agent as monotherapy, one patient (4.8 %) was treated with a combination of an investigational targeted agent and conventional cytotoxic agent (MEK inhibitor in combination with gemcitabine), and one patient (4.8 %) was treated with a cytotoxic agent as monotherapy (a hypoxia-activated cytotoxic prodrug). Main targets of these drugs according to the protocols include heat-shock protein 90 (HSP90), breakpoint cluster region-Abelson (BCR-ABL), PDGFR, activin receptor-like kinase 1 (ALK1), mammalian target of rapamycin (mTOR/p70S6), phosphoinositide-3 kinase (PI3K), hepatocyte growth factor (HGF/c-MET), MEK, tumor endothelial marker-1 (TEM-1), integrin, protein tyrosine kinase-2 (PTK2), VEGFR with MET, and FGFR.

**Table 1:** Patient characteristics

		*n* = 21	Percent
Age	Median (range)	57 (29–77)	-
Gender	Male	14	66.7
	Female	7	33.3
ECOG PS	0	19	90.5
	1	2	9.5
Primary site	Small intestine	10	47.6
	Stomach	7	33.3
	Others	4	19.0
Site of metastasis	Liver	20	95.2
	Peritoneum	10	47.6
	Lymph node	6	28.6
	Others	2	9.5
Number of metastases	0	1	4.8
	1–2	15	71.4
	≥3	5	23.8
Target lesion	Yes	21	100
Previous surgery	Yes	14	66.7
Number of previous chemotherapy	Median (range)	3 (2–5)	-
Previous treatment	Imatinib	21	100
	Sunitinib	20	95.2
	Regorafenib	9	42.9
Interval from first line chemotherapy to starting for phase I trials	Median (years)	4.5 (1.4–10.1)	-
Number of enrolled phase I trials	1	12	57.1
	≥2	9	42.9
Type of phase I trials	Targeting agents^a^	19	90.5
	Cytotoxic agents	1	4.8
	Targeting and cytotoxic agents	1	4.8

^a^Target: HSP90, BCR-ABL, PDGFR, ALK1, mTOR/p70S6, PI3K, HGF/c-MET, MEK, TEM-1, PTK2, VEGFR+MET, and FGFR

### Safety profiles

Safety profiles of the investigational drugs were evaluated in all 21 patients (Table 2). Four patients were excluded from DLT assessment cohort because of early disease progression. Thus, 17 patients (81.0 %) were assessed for DLT; none of the investigational drugs were associated with DLT. One patient (4.8 %) had SAE and was dead because of progressive disease. Two patients (9.5 %) experienced adverse events higher than grade 3; one patient developed neutropenia, whereas the other patient had increased serum creatine kinase levels. Two patients (9.5 %) discontinued protocol treatment during phase I clinical trials: one because of persistent grade 2 thrombocytopenia and the other because of vasculitis during infusion. One patient was receiving protocol treatment on the data cut-off day of March 2015. No treatment-related deaths were observed. One patient (4.8 %) died within 30 days after the last drug administration because of disease progression.

**Table 2:** Safety profiles

	*n* = 21	Percent
Dose-limiting toxicity (DLT)	0	0
Severe adverse events (SAE)	1	4.8
Death (due to progressive disease)	1	4.8
Grade 3 or higher toxicity	2	9.5
Blood system disorders	1	4.8
Laboratory abnormalities	1	4.8

### Tumor response

**Fig. 1:**
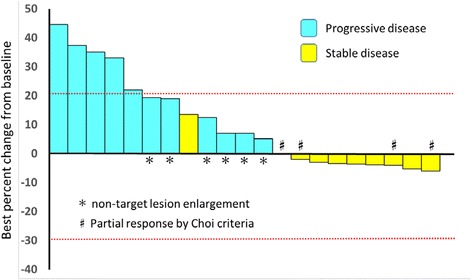
Waterfall plots of best response according to the RECIST

All patients were evaluated for tumor response. None of the 21 patients included in this study achieved CR or PR according to the RECIST. Ten patients (47.6 %) were determined as SD; four of them (19.0 %) maintained SD status for more than 24 weeks. Eleven patients (52.4 %) were judged as PD. Therefore, RR and DCR were 0 and 19.0 %, respectively. All four patients with SD more than 24 weeks were treated with targeted agents (BCR-ABL, ALK1, PI3K, and VEGFR with MET). However, we did not examine gene alternations in four patients with long SD. Therefore, we could not determine the relationship between the target gene alterations and targeted agents. A waterfall plot showed that eight patients (38.1 %) showed some shrinkage from the baseline (Fig. 1). Among them, four patients achieved PR according to the Choi criteria. Two cases were evaluated by PET. One patient with PR according to the Choi criteria showed significant reduction in FDG uptake [partial metabolic response (PMR): max SUV 33.5–24.4], and the other case evaluated as PD according to the RECIST showed increased FDG uptake and progressive metabolic response (PMD: max SUV 6.9–13.3) according to the EORTC PET response criteria [23].

### Survival analysis

**Fig. 2:**
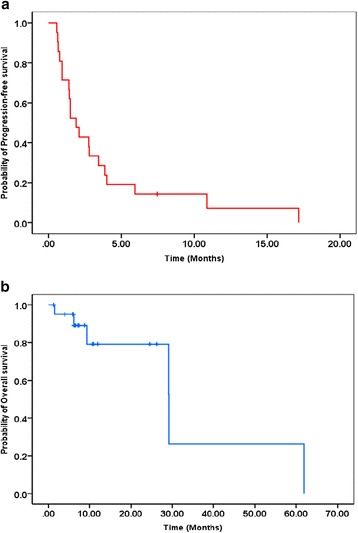
Kaplan–Meier plots of PFS and OS among all patients included in this study. **a** The median PFS of all patients was 1.9 months. **b** The median OS has not been reached

**Fig. 3:**
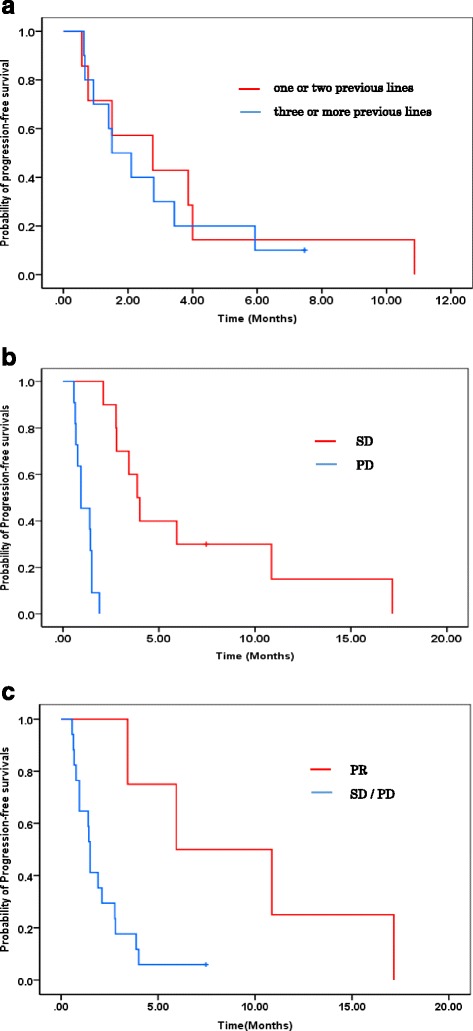
Kaplan–Meier plots of PFS according to the RESIST and Choi criteria. **a** PFS in patients who received one or two previous lines of therapy versus those who received three or more lines of therapy according to the RESIST. The PFS of patients who received one or two previous lines of chemotherapy (*n* = 7; median, 2.8 months) showed similar PFS as patients who received three or more lines (*n* = 10, median, 1.5 months; HR, 0.839; *P* = 0.739). **b** PFS of patients with SD and PD based on RESIST. Patients showing SD by RECIST (*n* = 10; median, 3.9 months) had significantly longer median PFS than those with PD (*n* = 11; median, 0.93 months; *P* < 0.0001). **c** PFS of patients with PR and SD/PD based on the Choi criteria. Patients showing PR according to the Choi criteria (*n* = 4; median, 5.9 months) also showed statistically longer PFS than the other patients (total *n* = 17; SD patients, *n* = 6 and PD patients, *n* = 11, 1.5 months; HR, 0.186; 95 % CI, 0.04–0.85; *P* = 0.03)

**Fig. 4:**
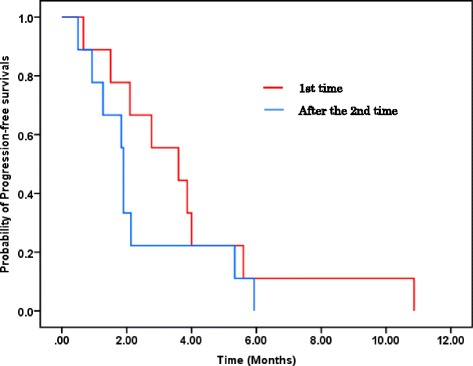
PFS of patients enrolled in two or more phase I clinical trials based on RECIST. We used the data obtained from nine patients who were enrolled in two or more phase I clinical trials. PFS of patients who underwent phase I clinical trial for the first time (*n* = 9; median, 3.6 months) showed similar PFS compared with patients who underwent phase I clinical trial thereafter (*n* = 9; median, 1.9 months; HR, 0.598; 95 % CI, 0.23–1.57; *P* = 0.297)

The overall median PFS in this study was 1.9 months, and the median OS has not yet been reached (Fig. 2a, b). Three-month PFS was estimated to be 33.3 %. Among a total of 21 patients, patients with one or two previous lines of chemotherapy (*n* = 7; median, 2.8 months) showed similar PFS to those with three or more lines (*n* = 10; median, 1.5 months; HR, 0.839; 95 % CI, 0.296–2.376; *P* = 0.739) (Fig. 3a). Patients showing SD according to the RECIST (*n* = 10; median, 3.9 months) had significantly longer median PFS than those showing PD (*n* = 11; median, 0.93 months; *P* < 0.0001) and patients showing PR according to the Choi criteria (*n* = 4; median, 5.9 months) also showed statistically longer PFS than the other patients (total *n* = 17; SD patients, *n* = 6 and PD patients, *n* = 11, 1.5 months; HR, 0.186; 95 % CI, 0.04–0.85; *P* = 0.03) (Fig. 3b and c). The efficacy of phase I clinical trials was compared among the first time phase I clinical trials and thereafter using the data obtained from nine patients who were enrolled in two or more phase I clinical trials, and there was no difference in the median PFS (Fig. 4).

## Discussion

In this study, we determined the clinical outcomes of GIST patients enrolled in phase I clinical trials and clarified values of phase I clinical trial for GIST patients who finished the standard chemotherapy and could not participate in disease-specific phase II or III clinical trials. Although phase I clinical trials are primarily designed to assess the safety and recommended dose of investigational agents [19], numerous trials evaluated and reported efficacy endpoints such as survival and tumor response [24], which may facilitate the expanded cohort and subsequent Phase II or III trials. Thus, not only safety evaluation but also overall clinical outcomes of each malignancy or mutation in phase I clinical trials may contribute to future development of drugs and clinical trials. To the best of our knowledge, this study is the first report on integrated analysis of GIST patients enrolled in multi-phase I clinical trials, although there have been a few reports on phase I clinical trials evaluating specific regimens for GIST patients, in which estimated PFS was between 1.5 and 4.8 months [25–27].

In this study, the safety profiles of investigational drugs were in agreement with the previously reported outcomes in patients with other malignancies [28, 29]. Two GIST patients (9.5 %) were withdrawn from a phase I clinical trial because of toxicity or treatment refusal, and there were no treatment-related deaths. In addition, death within 30 days after the last drug administration was observed in only one patient (4.8 %). These results suggested that the overall safety profiles were acceptable. Moreover, it is important to notice that nine patients (42.9 %) had participated in two or more phase I clinical trials by patients’ choice.

In this study, 47.6 % patients showed disease stabilization with the median PFS of 1.9 months in all patient populations and 3.9 months in patients who achieved SD by RECIST. These results were comparable to those reported by previous studies on imatinib rechallenge or TKIs beyond progression, which showed a DCR of 41–44.6 % and PFS of 1.8–2.4 months [15, 30, 31]. In this study, the 3-month progression-free rate was 33.3 %; the value appears to be marginal in terms of drug activities in the second-line therapy of sarcoma [32]. We recognized neither PR nor CR; however, in the third-line of sarcoma, responses were rarely observed in the early clinical trials. These results suggest that GIST patients may have some clinical benefits from participating in all comer phase I clinical trials with significant safety as previously reported in other malignancies [19–21, 23–28] and that phase I clinical trials may be one of treatment options for patients with GISTs refractory to the standard therapy.

Recent clinical trials in molecular targeted therapies showed significant efficacy even under the setting of phase clinical I trials. For example, BRAF inhibitors were shown to improve OS and PFS in patients with metastatic melanoma that had the BRAF V600E mutation [33]. More recently, phase I clinical trials aiming at immune checkpoint blockage with programmed death 1 (PD-1) or its ligand (PD-L1) antibodies showed higher response rates in several malignancies [34, 35]. Moreover, in the molecular-matching phase I trials between genetic aberration and targeting agents, matched patients had significant benefit, including ORR and OS, compared with the non-matched ones [36, 37]. Approximately 80 % of GIST patients have a mutation in *c-kit*, and 10 % harbor a mutation in *PDGFRA*. Therefore, development of TKIs of imatinib, sunitinib, and regorafenib targeting both KIT and PDGFRA tyrosine kinases is being vigorously advanced. However, activities of KIT-targeting drugs decreased with increased treatment lines. The wild-type GIST, nearly 10 % of GISTs and poor responders to imatinib, may have various genomic or epigenetic alterations in succinate dehydrogenase (SDH), neurofibromatosis type 1 (NF1), *BRAF*, or *RAS*. Molecular matching is suggested to be effective in SDH-deficient GISTs [38–40] and NF1-GISTs [41]. Therefore, profiling of genomic alterations may lead patients to right phase I clinical trials, although we lacked genomic information of GIST in this study.

There are several limitations in this study. Given the interval of 4.5 years from the first-line chemotherapy to enrollment in a phase I clinical trial, GIST patients in this study are a highly selected population, which may lead to selection bias. The small sample size, single-center population, and retrospective study may be limitations of this study. In this retrospective study, patient selection, patient choice, and patient-drug matching may be influenced by the availability of investigational drugs and investigators’ opinions.

## Conclusions

We showed that there were some clinical benefits for GIST patients who enrolled in phase I clinical trials, and overall safety profiles were acceptable. Our results suggest that after the failure of conventional chemotherapies, phase I trials might be one of treatment options for refractory GISTs.

## Abbreviations

BSC: Supportive care
CR: Complete response
CT: Computed tomography
DCR: Disease control rate
DLT: Dose-limiting toxicity
GISTs: Gastrointestinal stromal tumors
HR: Hazard ratios
MTD: Maximum-tolerated dose
ORR: Objective response rate
PDGFRA: Platelet-derived growth factor receptor alpha
PET: Positron emission tomography
PFS: Progression free survival
PR: Partial response
PS: Performance status
SAEs: Severe adverse events
SD: Stable disease
TTP: Time to progression

## References

[1] Tran T, Davila JA, El-Serag HB (2005). The epidemiology of malignant gastrointestinal stromal tumors: an analysis of 1,458 cases from 1992 to 2000. Am J Gastroenterol.

[2] Nishida T, Hirota S, Yanagisawa A, Sugino Y, Minami M, Yamamura Y (2008). Clinical practice guidelines for gastrointestinal stromal tumor (GIST) in Japan: English version. Int J Clin Oncol.

[3] Hirota S, Isozaki K, Moriyama Y, Hashimoto K, Nishida T, Ishiguro S (1998). Gain-of-function mutations of c-kit in human gastrointestinal stromal tumors. Science.

[4] Fletcher JA (2004). Role of KIT and platelet-derived growth factor receptors as oncoproteins. Semin Oncol.

[5] Corless CL, Heinrich MC (2008). Molecular pathobiology of gastrointestinal stromal sarcomas. Annu Rev Pathol.

[6] Miettinen M, Lasota J (2006). Gastrointestinal stromal tumors: review on morphology, molecular pathology, prognosis, and differential diagnosis. Arch Pathol Lab Med.

[7] Heinrich MC, Griffith DJ, Druker BJ, Wait CL, Ott KA, Zigler AJ (2000). Inhibition of c-kit receptor tyrosine kinase activity by STI 571, a selective tyrosine kinase inhibitor. Blood.

[8] Demetri GD, von Mehren M, Blanke CD, Van den Abbeele AD, Eisenberg B, Roberts PJ (2002). Efficacy and safety of imatinib mesylate in advanced gastrointestinal stromal tumors. N Engl J Med.

[9] Blanke CD, Demetri GD, von Mehren M, Heinrich MC, Eisenberg B, Fletcher JA (2008). Long-term results from a randomized phase II trial of standard- versus higher-dose imatinib mesylate for patients with unresectable or metastatic gastrointestinal stromal tumors expressing KIT. J Clin Oncol.

[10] Osusky KL, Hallahan DE, Fu A, Ye F, Shyr Y, Geng L (2004). The receptor tyrosine kinase inhibitor SU11248 impedes endothelial cell migration, tubule formation, and blood vessel formation in vivo, but has little effect on existing tumor vessels. Angiogenesis.

[11] Mendel DB, Laird AD, Xin X, Louie SG, Christensen JG, Li G (2003). In vivo antitumor activity of SU11248, a novel tyrosine kinase inhibitor targeting vascular endothelial growth factor and platelet-derived growth factor receptors: determination of a pharmacokinetic/pharmacodynamics relationship. Clin Cancer Res.

[12] Shirao K, Nishida T, Doi T, Komatsu Y, Muro K, Li Y (2010). Phase I/II study of sunitinib malate in Japanese patients with gastrointestinal stromal tumor after failure of prior treatment with imatinib mesylate. Invest New Drugs.

[13] Demetri GD, van Oosterom AT, Garrett CR, Blackstein ME, Shah MH, Verweij J (2006). Efficacy and safety of sunitinib in patients with advanced gastrointestinal stromal tumour after failure of imatinib: a randomised controlled trial. Lancet.

[14] Demetri GD, Reichardt P, Kang YK, Blay JY, Rutkowski P, Gelderblom H (2013). Efficacy and safety of regorafenib for advanced gastrointestinal stromal tumours after failure of imatinib and sunitinib (GRID): an international, multicentre, randomised, placebo-controlled, phase 3 trial. Lancet.

[15] Kang YK, Ryu MH, Yoo C, Ryoo BY, Kim HJ, Lee JJ (2013). Resumption of imatinib to control metastatic or unresectable gastrointestinal stromal tumours after failure of imatinib and sunitinib (RIGHT): a randomised, placebo-controlled, phase 3 trial. Lancet Oncol.

[16] von Mehren M, Benjamin RS, Bui MM, Casper ES, Conrad EU, DeLaney TF (2012). Soft tissue sarcoma, version 2.2012: featured updates to the NCCN guidelines. J Natl Compr Canc Netw.

[17] ESMO/European Sarcoma Network Working Group (2014). Gastrointestinal stromal tumours: ESMO Clinical Practice Guidelines for diagnosis, treatment and follow-up. Ann Oncol.

[18] Japan Society of Oncology GIST: gastrointestinal stromal tumor. http://www.jsco-cpg.jp/item/03/index.html 10.2169/naika.93.1451 15298286

[19] Agrawal M, Emanuel EJ (2003). Ethics of phase 1 oncology studies: reexamining the arguments and data. JAMA.

[20] Kawazoe A, Shitara K, Fukuoka S, Noguchi M, Kuboki Y, Bando H (2015). Clinical outcomes in 66 patients with advanced gastric cancer treated in phase I trials: the NCCHE experience. Invest New Drugs.

[21] Tsimberidou AM, Vaklavas C, Wen S, Hong D, Wheler J, Ng C (2009). Phase I clinical trials in 56 patients with thyroid cancer: the M. D. Anderson Cancer Center experience. J Clin Endocrinol Metab.

[22] Young H, Baum R, Cremerius U, Herholz K, Hoekstra O, Lammertsma AA (1999). Measurement of clinical and subclinical tumor response using [18F]-fluorodeoxyglucose and positron emission tomography: review and 1999 EORTC recommendations. Eur J Cancer.

[23] Choi H, Charnsangavej C, Faria SC, Macapinlac HA, Burgess MA, Patel SR (2007). Correlation of computed tomography and positron emission tomography in patients with metastatic gastrointestinal stromal tumor treated at a single institution with imatinib mesylate: proposal of new computed tomography response criteria. J Clin Oncol.

[24] Topalian SL, Sznol M, McDermott DF, Kluger HM, Carvajal RD, Sharfman WH (2014). Survival, durable tumor remission, and long-term safety in patients with advanced melanoma receiving nivolumab. J Clin Oncol.

[25] Bauer S, Hilger RA, Mühlenberg T, Grabellus F, Nagarajah J, Hoiczyk M (2014). Phase I study of panobinostat and imatinib in patients with treatment-refractory metastatic gastrointestinal stromal tumors. Br J Cancer.

[26] Wagner AJ, Chugh R, Rosen LS, Morgan JA, George S, Gordon M (2013). A phase I study of the HSP90 inhibitor retaspimycin hydrochloride (IPI-504) in patients with gastrointestinal stromal tumors or soft-tissue sarcomas. Clin Cancer Res.

[27] Demetri GD, Casali PG, Blay JY, von Mehren M, Morgan JA, Bertulli R (2009). A phase I study of single-agent nilotinib or in combination with imatinib in patients with imatinib-resistant gastrointestinal stromal tumors. Clin Cancer Res.

[28] Cassier PA, Polivka V, Judson I, Soria JC, Penel N, Marsoni S (2014). Outcome of patients with sarcoma and other mesenchymal tumours participating in phase I trials: a subset analysis of a European Phase I database. Ann Oncol.

[29] Roberts TG, Goulart BH, Squitieri L, Stallings SC, Halpern EF, Chabner BA (2004). Trends in the risks and benefits to patients with cancer participating in phase 1 clinical trials. JAMA.

[30] Sawaki A, Kanda T, Komatsu Y, Nishida T (2014). Impact of rechallenge with imatinib in patients with advanced gastrointestinal stromal tumor after failure of imatinib and sunitinib. Gastroenterol Res Pract.

[31] Reichardt P, Blay JY, Gelderblom H, Schlemmer M, Demetri GD, Bui-Nguyen B (2012). Phase III study of nilotinib versus best supportive care with or without a TKI in patients with gastrointestinal stromal tumors resistant to or intolerant of imatinib and sunitinib. Ann Oncol.

[32] Van Glabbeke M, Verweij J, Judson I, Nielsen OS (2002). Progression-free rate as the principal end-point for phase II trials in soft-tissue sarcomas. Eur J Cancer.

[33] Chapman PB, Hauschild A, Robert C, Haanen JB, Ascierto P, Larkin J (2011). Improved survival with vemurafenib in melanoma with BRAF V600E mutation. N Engl J Med.

[34] Brahmer JR, Tykodi SS, Chow LQ, Hwu WJ, Topalian SL, Hwu P (2012). Safety and activity of anti-PD-L1 antibody in patients with advanced cancer. N Engl J Med.

[35] Topalian SL, Hodi FS, Brahmer JR, Gettinger SN, Smith DC, McDermott DF (2012). Safety, activity, and immune correlates of anti-PD-1 antibody in cancer. N Engl J Med.

[36] Tsimberidou AM, Iskander NG, Hong DS, Wheler JJ, Falchook GS, Fu S (2012). Personalized medicine in a phase I clinical trials program: the MD Anderson Cancer Center initiative. Clin Cancer Res.

[37] Tsimberidou AM, Wen S, Hong DS, Wheler JJ, Falchook GS, Fu S (2014). Personalized medicine for patients with advanced cancer in the phase I program at MD Anderson: validation and landmark analyses. Clin Cancer Res.

[38] Gill AJ (2012). Succinate dehydrogenase (SDH) and mitochondrial driven neoplasia. Pathology.

[39] Ganjoo KN, Villalobos VM, Kamaya A, Fisher GA, Butrynski JE, Morgan JA (2014). A multicenter phase II study of pazopanib in patients with advanced gastrointestinal stromal tumors (GIST) following failure of at least imatinib and sunitinib. Ann Oncol.

[40] George S, Wang Q, Heinrich MC, Corless CL, Zhu M, Butrynski JE (2012). Efficacy and safety of regorafenib in patients with metastatic and/or unresectable GI stromal tumor after failure of imatinib and sunitinib: a multicenter phase II trial. J Clin Oncol.

[41] Jessen WJ, Miller SJ, Jousma E, Wu J, Rizvi TA, Brundage ME (2013). MEK inhibition exhibits efficacy in human and mouse neurofibromatosis tumors. J Clin Invest.

